# Nanosystems for chemodynamic based combination therapy: Strategies and recent advances

**DOI:** 10.3389/fphar.2022.1065438

**Published:** 2022-10-28

**Authors:** Minghui Li, Wen Zhang, Xiaopeng Xu, Guoying Liu, Mengfei Dong, Kaoxiang Sun, Peng Zhang

**Affiliations:** School of Pharmacy, Key Laboratory of Molecular Pharmacology and Drug Evaluation (Yantai University), Ministry of Education, Collaborative Innovation Center of Advanced Drug Delivery System and Biotech Drugs in Universities of Shandong, Yantai University, Yantai, China

**Keywords:** chemodynamic therapy (CDT), nanosystems, combination therapy, Fenton reaction, reactive oxygen species

## Abstract

Chemodynamic therapy (CDT), a newly developed approach for cancer treatment, can convert hydrogen peroxide (H_2_O_2_) into toxic hydroxyl radicals (•OH) by using Fenton/Fenton-like reaction to kill tumor cells. However, due to the complexity of the intracellular environment of tumor cells, the therapeutic efficacy of CDT was severely restricted. Recently, combination therapy strategies have become popular approaches for tumor treatment, and there are numerous studies have demonstrated that the CDT-based combination strategies can significantly improve the anti-tumor efficiency of CDT. In this review, we outline some of the recent progress in cancer chemodynamic therapy from 2020, and discuss the progress in the design of nanosystems for CDT synergistic combination therapies.

## Introduction

Currently, malignant tumor has become a major threat to human health, considerable attention has been paid to explore more effective therapeutic strategies for cancer treatment (Cao M. et al., 2020; Ferlay et al., 2021; Sung et al., 2021). With the development of nanoscience and nanobiotechnology, significant progress has been achieved in cancer therapy in the last decades (Wicki et al., 2015; Lin et al., 2022). However, effective treatment for cancer still presents significant challenges, for example, multidrug resistance is one of the difficult issues in current clinical chemotherapy treatment. Therefore, it is particularly necessary to develop new therapeutic agents and approaches for the more satisfactory cancer treatment (Rasool et al., 2022). In recent years, chemodynamic therapy (CDT) has attracted extensive attention in the field of tumor therapy.

**FIGURE 1:**
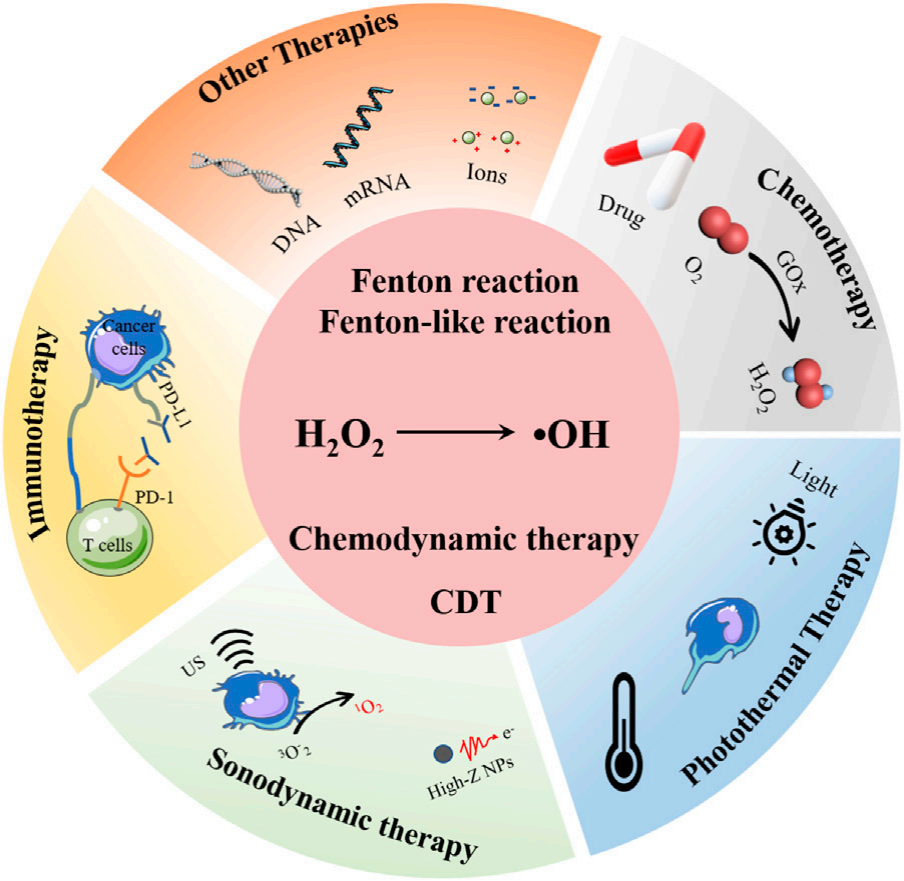
CDT and CDT-based combined therapy strategies, including combination with chemotherapy, photothermal therapy, sonodynamic therapy, and immunotherapy.

CDT is inherently oxidative stress-induced cell killing process, it was first proposed in 2016 (Zhang et al., 2016). Briefly, by using the transition metal ions (Fe^2+^, Cu^+^, Mn^2+^, and so on), intracellular hydrogen peroxide (H_2_O_2_) is catalyzed to generate high reactivity hydroxyl radical (•OH), which would induce oxidative stress and further the death of cancer cells (Zhang X. et al., 2020; Chen et al., 2020; Zhang H. et al., 2021) (Figure 1). Generally, in order to improve the specificity and therapeutic efficacy of CDT, those CDT agents are usually designed as nanosystems. In this review, recent advancements of nanosystems for enhancing the CDT application and efficiency are reviewed. The major obstacles for the clinical applications of CDT are introduced, and those CDT enhanced strategies that CDT in combination with other therapies are focused and emphasized, such as combined with photothermal therapy, immunotherapy, sonodynamic therapy, and so on. The present review aims to provide better understanding of how to design effective chemodynamic agents, thus to broaden the application of CDT and maximize the CDT therapeutic effectiveness.

## Chemodynamic therapy

It is well known that the tumor microenvironment (TME) exhibits unique physiological features compare with the normal tissues, including acidic pH, hypoxia, overproduced H_2_O_2_, the alteration of specific enzyme activities and elevated level of glutathione (GSH) (Roma-Rodrigues et al., 2019; Zhang Y. et al., 2022). On one side, these features of TME play crucial roles in tumor initiation, progression, metastasis, and even resistance to therapy. On the other side, the unique physiological conditions of TME can also be used as the stimuli to trigger the antitumor drug release at tumor sites to improve antitumor efficacy, which are known as the TME stimuli-responsive drug delivery systems (Wu and Dai, 2017; Li et al., 2021).

Considering the acidic and H_2_O_2_ overexpressed conditions in TME, the concept of CDT was proposed as a therapeutic strategy that using Fenton or Fenton-like reaction to generate hydroxyl radical in tumor site. In the first report of CDT, ferrous ions (Fe^2+^) was released from iron-based nanomaterials, which could catalyze endogenous H_2_O_2_ decomposition into •OH under acidic conditions of TME (Fe^2+^ + H_2_O_2_ → Fe^3+^ + •OH + OH^−^, known as Fenton reaction). The over produced •OH, one type of cytotoxic reactive oxygen species (ROS), had strong oxidative activity and caused severe oxidative damage to induce tumor apoptosis by phospholipid peroxidation, mitochondrial dysfunctions, and DNA damage (Khan et al., 2012; Srinivas et al., 2019).

Besides Fenton reaction, Fenton-like reactions are also employed for CDT application. Not restricted to iron, other reactive metal components, such as Cu^+^, Mn^2+^, Mo^6+^, are used in Fenton-like reaction to catalyze the generation of •OH from H_2_O_2_ (Cao et al., 2019, Cao et al., 2020 S.; Liang et al., 2021, 2022; Lin et al., 2021). It should be noted that Fenton reaction highly relies on the overexpression of H_2_O_2_ and acidic condition of TME, which makes CDT strategy much safer to the normal tissues, since the Fenton reaction was suppressed under the neutral and insufficient H_2_O_2_ conditions in the healthy cell microenvironment.

## Challenges and limitations of CDT

As a type of ROS-mediated cancer treatment modality, there is considerable development of understanding and in CDT during the last years. However, there are still challenges and limitations that hinder its further application in clinical. First, the acidic environment of solid tumor site is insufficient to trigger the efficient Fenton/Fenton-like reaction; second, the endogenous H_2_O_2_ level in tumor cells is not enough to generate adequate •OH to induce apoptosis of tumor cells; third, the high concentration of reduced glutathione and redox homeostasis of tumor cells could eliminate the free radical and weaken the CDT effect.

From the perspective of chemical kinetic, hydroxyl radicals could be catalyzed *via* Fenton/Fenton-like reaction over a wide range of pH conditions (between acidic and neutral pH) (Zhu et al., 2019). However, the efficiency and reactivity of Fenton reaction strongly depend on the pH condition, the reactivity is obviously increased in the acidic pH range (pH 2–4), and strongly reduced (even lost) in alkaline media. Considered the TME is slightly acidic pH condition (pH 6.5–6.8), at one side, the limited reactivity of Fenton/Fenton-like reaction in healthy tissues (pH 7.2–7.4) could avoid the damage on healthy tissues and cells, which makes CDT might selectively kill tumor cells; on the other side, the weakly acidic TME limits the efficiency of Fenton/Fenton-like reactions, which significantly reduce the generation of •OH.

To address the problem of insufficient acidity, attempts have been made to reconstitute the tumor microenvironment *via in situ* H^+^ generation strategy. For example, several studies have integrated glucose oxidase (GOx) into CDT agents, since GOx can oxidize glucose to gluconic acid to further increases acidity at TME, thus could enhance Fenton/Fenton-like reaction efficiency (Cheng et al., 2020; Zhang X. et al., 2021). It should be noted that the tumor cells employ various mechanisms to maintain cellular homeostasis during tumorigenesis and metastasis, this property makes the *in situ* acidization of intracellular strategy couldn’t support a long-term change. Therefore, the design and development of pH-independent CDT nanotherapeutics and/or combinational therapies with CDT are viable approaches to overcome pH limitations of CDT.

Besides the pH condition, Fenton/Fenton-like systems are also H_2_O_2_ dependent. Compared to normal cells (extremely low H_2_O_2_ level), the tumor cells produce and accumulate a large amount of H_2_O_2_ (100 μM–1 mM). However, the initial endogenous H_2_O_2_ level in tumor cells was still not enough to generate sufficient •OH to achieve satisfactory antitumor therapeutic effect. In addition, there are endogenous reducing substances that are involved in maintaining cellular redox homeostasis in the tumor cells. These reductive substances, for example reduced glutathione, could mitigate oxidative stress and repair the oxidative damage. Therefore, increasing H_2_O_2_ concentration and reducing GSH level in tumor cells are potential approaches to enhance the efficacy of CDT. To overcome the limited-H_2_O_2_-level obstacle, tremendous H_2_O_2_ self-supplying systems are designed to enhance the efficiency of Fenton/Fenton-like reactions to achieve satisfactory CDT effect. From the reported studies, there are two important design roles for constructing these H_2_O_2_ self-supplying systems: *1*) delivery of exogenous H_2_O_2_ to tumors (Gao et al., 2019; Han et al., 2020; Zhao et al., 2022); *2*) promote the ability of endogenous H_2_O_2_ production (Liu et al., 2020; Sang et al., 2020; Jiang et al., 2022).

## Nanosystems for CDT based combination therapy strategies

With the rapid expansion of chemistry and materials science, nanotechnology shows great potential to overcome the limitations of conventional tumor therapy, and nanosystems (or nanotherapeutics) are regarded as one of the most promising strategies for better tumor treatment (Zhang P. et al., 2022). The recent reports have revealed that the combination therapy strategies with CDT nanosystems can optimize the therapy effects and minimize the toxicity and adverse effects of treatment (He et al., 2021; Yang et al., 2021; Liu Y. et al., 2022; Hao et al., 2022).

### CDT-chemotherapy combination therapy

Although there is tremendous progress in cancer therapy during last years, chemotherapy is still main therapeutic modality in clinic treatment of tumor. However, the low targeting activity, multidrug resistance problems, and associated serious adverse-effects (for example myelosuppression) are still the major challenges and limitation in the development of effective chemotherapeutic agents (Holohan et al., 2013; Javarappa et al., 2018). Therefore, the combinational therapy strategy has become the potential cancer treatment. For example, the synergistic anti-tumor strategy of CDT combined with chemotherapy shows excellent prospects for tumor treatment since the chemotherapy can decrease GSH level in tumor cells. Furthermore, specifically activated by the stimuli (such as pH) in TME, CDT-chemotherapy combination therapy could solve the drug-resistance problem and significantly enhance the anti-tumor efficacy (Tan et al., 2020; Xiao et al., 2021; Yao et al., 2021).

Doxorubicin (DOX), a commonly used chemotherapy drug, has been demonstrated that has the ability to regulate the generation of intracellular H_2_O_2_ and GSH. Recent studies have revealed that the synergistic enhanced therapy by combining CDT with DOX. Mu reported a nanosystem prepared from epigallocatechin gallate (EGCG) and Fe^3+^ to simultaneously deliver DOX and iron ions to tumor sites. DOX/Fe^3+^/EGCG nanoparticles exhibit good stability, and response to the high level of glutathione and acidic conditions in TME to achieve efficient release of DOX and Fe^3+^ after internalization. After Fe^3+^ was reduced to Fe^2+^ by EGCG, Fenton reaction was induced to produce •OH, which further induced tumor death to amplify the therapeutic effect of DOX. *In vitro* and *in vivo* studies have also shown the promising tumor treatment results by this CDT-chemotherapy combined system (Mu et al., 2020). Huang’s group constructed a biodegradable multifunctional copper-doped calcium phosphate nanocomposite system to incorporate DOX. Due to the glucose oxidase, this nanocomposite catalyzed glucose to produce gluconic acid and hydrogen peroxide, and Cu^2+^ was reduced to Cu^+^ by endogenous GSH. This strategy could consume GSH and enhance H_2_O_2_ at the same time, thus to amplify CDT effect. Within tumor cells, Cu^2+^/Cu^+^ induced the Fenton-like reaction to increase •OH level, enhancing the chemotherapy results. In addition, the DOX in this system can not only be used for chemotherapy, but also for fluorescence imaging to monitor of tumor targeting and drug release in real-time due to its inherent fluorescence property (Fu L. H. et al., 2021).

### CDT-immunotherapy combination therapy

Immunotherapy is an emerging field in tumor treatment. Accumulating evidence indicates that the engagement of CD8^+^ T lymphocytes and nature killer cells in TME is the critical process for successful immunotherapy (Wang et al., 2019). Recent studies have shown that reactive oxygen species play a vital role in inducing the immunogenic death of tumor cells and facilitating antitumor immune responses. Immunogenic cell death induced by ROS oxidative stress could increase the release of inflammatory cytokines, such as tumor necrosis factor α (TNFα), which could further activate the immune response *in vivo* to inhibit and eliminate both primary tumor cells and distant sites tumor (or secondary cancer cells) (Fu L. et al., 2021; Huang et al., 2021).

Li and Rong reported a novel nanosystem by loading ultra-small CaO_2_ and Fe_3_O_4_ nanoparticles into dendritic mesoporous silica which coated with a pH-responsive membrane. This nanocomposite realized the synergism of CDT and immunotherapy for effective cancer therapy. After intravenous administration of this nanocomposite, acidic condition of TME triggered CaO_2_ to generate abundant H_2_O_2_, which was catalyzed to produce •OH through Fenton reaction mediated by Fe_3_O_4_. By inducing tumor cells death and the release of tumor-associated antigens, the tumor immunogenic microenvironment was altered and immune responsibility was enhanced by increasing the rates of CD8^+^ Treg cells and M1/M2 macrophage. This study provides a feasible strategy to achieve highly effective cancer treatment through the synergistic effect of CDT-immunotherapy (Li and Rong, 2020).

### CDT-photothermal combination therapy

Photothermal therapy (PTT) is a unique cancer therapeutic strategy, it employs photo-absorbing agents to convert the light energy into thermal energy, and the heat generated through photo-thermal reaction is utilized to directly destroy tumor tissues (Doughty et al., 2019; Zhao et al., 2021). However, PTT usually relies on a high-energy light source for long term treatment, and it is difficult to achieve satisfactory therapeutic effect with only PTT one single modal therapy.

By hybridizing Fenton agents, PTT systems can be designed to combine with chemodynamic therapy. In the combination system, the heat generated by photo-thermal reaction can not only effectively kill tumor cells, but also increase the rate of ROS production, which ultimately enhanced the effect of chemodynamic therapy (Zhang M. et al., 2020; Jiang et al., 2020; Shi et al., 2022). Li *et al.* designed a biomimetic nanosystem for self-enhanced photothermal/chemodynamic synergistic therapy. In this study, a nanoplatform was constructed by loading glucose oxidase (GOD) and Ag_2_S quantum dots into MnO_2_ nanosheets and then coated with the 4T1 cell membrane, inducing successfully escape immune clearance. This nanoplatform exhibited good tumor targeting ability and biocompatibility. In addition to the photothermal treatment effect, glucose oxidase (GOD) can oxidize excess glucose to H_2_O_2_, and at the same time, the released Mn^2+^ can catalyze H_2_O_2_ to generate abundant •OH through Fenton-like reaction. Therefore, photothermal enhanced chemodynamic therapy can be achieved under near-infrared light irradiation (Li et al., 2022).

### CDT-sonodynamic combination therapy

Sonodynamic therapy (SDT) has been developed as a promising noninvasive approach for tumor treatment in recent decades. The antitumor mechanism of SDT is ROS-based process, briefly, a sonosensitizer is activated by ultrasound energy to generate ROS that destroy the tumor cells (Gong and Dai, 2021; Geng et al., 2022; Sun et al., 2022). However, SDT also has some problems, such as low ROS yield, limited delivery efficiency and short/long-term safety concerns of sonosensitizers. There is a two-dimensional nanosonosensitizer/nanocatalyst combined nanosystem reported by Tang’s group. In this nanosystem, integrated Cu_2_O could promote the *in situ* generation of H_2_O_2_ in the acidic tumor microenvironment, and the generated H_2_O_2_ further oxidized Ti_3_C_2_ to TiO_2_, which was the nanosonosensitizer and reacted with water and oxygen into the cells to generate ROS. In addition, ultrasound also enhances the Cu-induced Fenton-like reaction during the sonodynamic process to generate more ROS to achieve sonodynamic/chemodynamic improved synergistic tumor therapy. This study also confirmed that the antitumor mechanism of synergistic chemodynamic and sonodynamic therapies are associated with the upregulation of oxidative phosphorylation and ROS generation (Zhang M. et al., 2022).

In addition to the above-mentioned combination therapy strategies, CDT can be also applied with other therapy to achieve the synergistic effects and enhanced therapeutic effects. Lin *et al.* constructed a hyaluronic acid-modified bimetallic peroxide CaO_2_-CuO_2_@HA nano-system, which was effectively accumulated at the tumor site through the EPR effect, and the modified hyaluronic acid could recognize CD44 on the surface of tumor cells, which achieved active targeted property. Subsequently, the nanocomposites are able to generate a large number of Ca^2+^, Cu^2+^, and hydrogen peroxide in the acidic and hyaluronidase-overexpressing tumor microenvironment. Accompanied with glutathione depletion, Cu^2+^ and H_2_O_2_ induced Fenton-like reaction to generate more •OH. In addition, the excess Ca^2+^ released from the nanosystem leaded to the mitochondrial damage, which further enhanced oxidative stress in tumor cells. In addition, the imbalance of calcium transport channels caused by oxidative stress further promoted the calcification, inducing the necrosis of tumor cells (also named as ion interference therapy). Therefore, during ROS generation process, the Fenton-like reaction generated by Cu^2+^ is synergistic with the mitochondrial dysfunction induced by Ca^2+^ (Liu B. et al., 2022).

## Conclusion and prospects

Although it has been proven that CDT is an ideal therapeutic approach for tumor therapy, the intrinsic barrier of TME is the main obstacle to hinder the further development and clinical translation of CDT. Fortunately, with the rapid expansion of nanoscience and nanobiotechnology, there are more nanosystems have been designed and studied that have potential to overcome those challenges of CDT. However, there are still some problems need to be solved before further clinical applications:

(1) Biosafety issues. At the cellular level, CDT-based nanosystems can enter cells in a variety of approaches, which may lead to changes or even loss of normal cellular functions, resulting in unnecessary toxic side effects. Especially, the current reported CDT-based nanosystems are mainly inorganic or hybrid nanomaterials, which are liable to elicit *in vivo* immune response. Thus, the biosafety issue of CDT-based nanosystems has raised intense concerns in the clinical applications.
(2) Complexity of CDT-based nanosystems. In the present studies, CDT-based nanosystems are designed overly complicated but hardly used in clinical practice. On one side, complicated nanosystems usually are associated to biological toxicity since the chemical compositions are too complex to precisely predict the biocompatibility. On the other side, the synthesis of CDT-based nanosystems is only reported in the lab level, but for practical applications, the reproducibility is too low for industrial scale. Therefore, how to design and construct CDT-based nanosystems with simple structure, stable compositions, and efficient responsibility to endogenous and/or exogenous stimuli has attracted much attention.
(3) The synergistic mechanism of CDT-based combined strategies. CDT and CDT-based combined strategies are ROS-mediated process, however, the mechanism for •OH production by Fenton/Fenton-like reactions *in vivo* need be deeply investigated, especially the CDT-based combined strategies. A successful combined therapy can overcome the shortcomings of monotherapy, however, synergistic therapy may provide new therapeutic mechanism and opportunities for tumor therapy. To further broaden the application of CDT and CDT-based combined therapy, ROS synergistic mechanism *in vivo* should be deeply investigated to provide more effective therapeutic options.

In the future, research efforts should shift to practical trials of using CDT and CDT-based combined therapy strategies in disease treatment. There is still a long way to go before achieving clinical application, we expect more nanosystems for CDT therapy are designed and more cancer patients will benefit from this treatment.
